# Cariprazine augmentation in patients with treatment resistant unipolar depression who failed to respond to previous atypical antipsychotic add-on. A case-series

**DOI:** 10.3389/fpsyt.2023.1299368

**Published:** 2023-11-27

**Authors:** Enrico Pessina, Azzurra Martini, Fabiola Raffone, Vassilis Martiadis

**Affiliations:** ^1^Department of Mental Health, ASL Cuneo 2, Bra, Italy; ^2^Department of Mental Health, ASL Napoli 1 Centro, Napoli, Italy

**Keywords:** major depression, treatment resistance, cariprazine, augmentation, atypical antipsychotic

## Abstract

Among individuals receiving an adequate pharmacological treatment for Major Depressive Disorder (MDD), only 30% reach a full symptom recovery; the remaining 70% will experience either a pharmacological response without remission or no response at all thus configuring treatment resistant depression (TRD). After an inadequate response to an antidepressant, possible next step options include optimizing the dose of the current antidepressant, switching to a different antidepressant, combining antidepressants, or augmenting with a non-antidepressant medication. Augmentation strategies with the most evidence-based support include atypical antipsychotics (AAs). Few data are available in literature about switching to another antipsychotic when a first augmentation trial has failed. We present a case-series of patients with unipolar treatment resistant depression who were treated with a combination of antidepressant and low dose of cariprazine after failing to respond to a first augmentation with another AA. We report data about ten patients affected by unipolar depression, visited at the outpatients unit of Mental Health Department of ASL CN2 of Bra and NA1 of Napoli (Italy). All patients failed to respond to conventional antidepressant therapy. A low dose of AA (aripiprazole, risperidone or brexpiprazole) was added for one month to the ongoing antidepressant treatment without clinical improvement. A second augmentation trial was then made with cariprazine. Seven out of ten patients were responders at the end of period, of them 1 patient reached responder status by week 2. HAM-D mean scores decreased from 23.9 ± 3.9 (baseline) to 14.8 ± 5.3 (4 weeks). Cariprazine was well tolerated, no severe side effect was observed during the trial. Our sample of treatment resistant unipolar patients showed good response to augmentation with cariprazine. Failure to a first AA-augmentation trial does not preclude response to a second one. This preliminary result requires confirmation through more rigorous studies conducted over greater samples.

## Introduction

Despite the availability of many pharmacological treatment options, nearly about a half of patients affected by Major Depression Disorder (MDD) do not adequately responds to antidepressant (AD) treatment (1, 2). Treatment resistant depression (TRD) is a serious and disabling illness with significant impact on social and occupational outcomes (3). Current strategies to treat patients who do not respond to first-line antidepressant monotherapy include switching AD (either within or between classes) or combining different drugs (4). After failure of 2 AD treatments, current guidelines indeed suggest augmentation strategies (5). Effective agents to add on to ongoing AD, according to literature, could be chosen between mood stabilizers, ADs, thyroid hormones, ketamine or atypical antipsychotic (AA) (5–7). Aripiprazole (8, 9), olanzapine (10, 11), quetiapine (12, 13) and risperidone (14, 15) showed efficacy in augmentation trial for patients affected by TRD. More recently brexpiprazole (16–18) and cariprazine (19–21) also demonstrated their efficacy for TRD. Not withstanding various studies that show efficacy of AAs as dd-on strategy to ameliorate depressive symptoms in TRD, there is a lack of literature, to our knowledge, about efficacy of a second trial with an AA in those patients who failed to respond to a first augmentation trial with antipsychotic. We report a case series of TRD patients who failed to respond to an augmentation with a first AA to their ongoing AD and were subsequently treated with low dose cariprazine (CPZ) as add-on. Cariprazine is a partial agonist of dopamine D2/D3 receptors (preferring D3) and serotonin 5HT1A/5Ht2A receptors (22). This unique receptor profiles may play a role in its efficacy and tolerability and are believed to be involved in the antipsychotic, antidepressant, antianhedonic and pro-cognitive effects (23, 24). FDA has approved cariprazine as an adjunctive treatment for unipolar depression (1.5–3 mg/day) however in Europe it has been approved only for schizophrenia (25).

## Materials and methods

Clinical records of inpatients and outpatients with a diagnosis of Major Depressive Disorder according to DSM-5 criteria treated in the Mental Health Department of Alba and Bra (Italy) and Mental Health Department of Napoli 1 (Italy) from July 2022 and March 2023 were analyzed. All patients presented with some form of treatment resistance that was defined according to operational criteria provided by Sourey et al. (26). All patients were treated with a AA (aripiprazole, risperidone or brexpiprazole) added to ongoing AD therapy for 4 weeks without response estimated as reduction of Hamilton Depression Rating Scale (HAM-D) (27) score of at least 50% from the beginning of the augmentation. After a wash-out period from the first AA of 2 weeks maintaining the ongoing AD treatment unchanged, patients underwent a second augmentation trial of 4 weeks with cariprazine. Cariprazine starting dose was 1.5 mg/day for all patients. Dosage changes were established according to clinical judgment (no specific guidelines were followed. Dosage variation was established according to efficacy observed and tolerability). AD dose was maintained unchanged during the weeks of add-on.

All subjects referred to our Service did sign a written informed consent to have their clinical data potentially used for teaching or search purposes, anonymously treated. Written consent was also collected for off-label treatment. Socio-demographic, clinical and safety information were collected for each subject from medical reports. Patients underwent control visits according to clinical practice. All psychiatric diagnoses and clinical assessment were made by psychiatrist with several years of experience. Due to the frequent presence of bipolar spectrum features in TRD patients, careful screening was made by psychiatrist for this diagnosis also by mean of Mood Disorder Questionnaire (MDQ). For the purpose of this report, medical records have been analyzed at the start of treatment with cariprazine, after 2 weeks and after 4 weeks. Clinical symptoms of depression were assessed by means of HAM-D. The effectiveness of cariprazine was assessed evaluating the change of HAM-D scores from baseline to endpoint (4 weeks). Due to exiguity of the sample no statistical analysis was performed.

## Results

**Figure 1:**
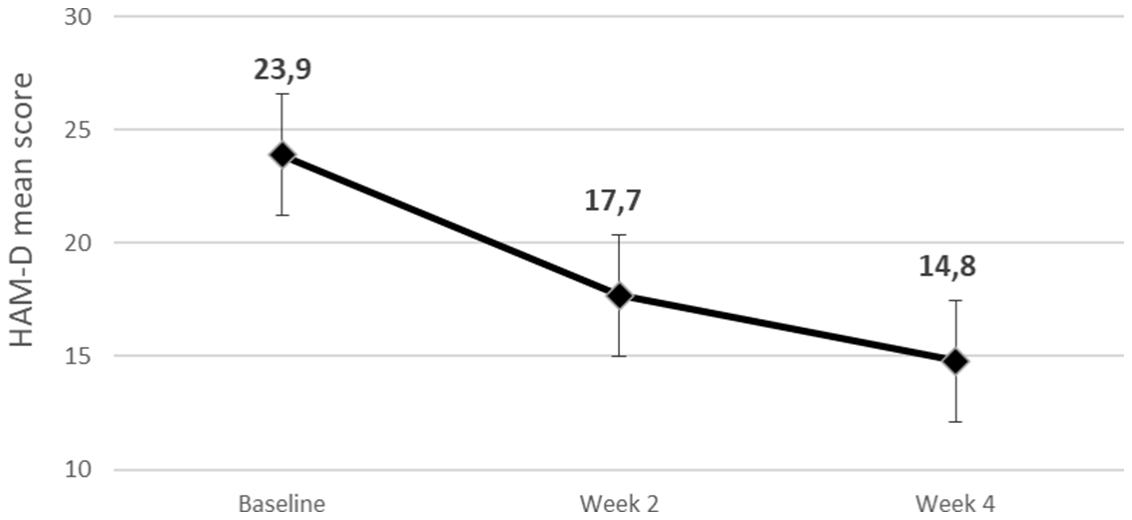
Mean reduction of Hamilton Depression Rating Scale (HAM-D) scores during the 4 weeks observation period.

We report on a case series of 10 patients. 6 patients (60.0%) were female. The mean age of the sample was 52.3 ± 6.2 years. The mean age at onset of Major depressive disorder was 25.4 ± 4.1 years. 4 patients (40.0%) had at least one suicidal attempt lifetime. About two-thirds of patients (60%) had other comorbid psychiatric disorders. All socio-demographic and clinical characteristics of the patients are shown in Table 1, including the AA used in the first augmentation trial. Mean doses of antipsychotic in the first trial were, respectively, 4.4 ± 1.2 mg/day for aripiprazole, 1 ± 0 mg/day for brexpiprazole and 0.8 ± 2.3 mg/day for risperidone (risperidone in add on ranged from 0.5 to 1.5 mg/day). Table 2 reports duration of the single episode of treatment resistant depression, the AD combined with cariprazine and its dosage. All patients completed the 4 weeks period of cariprazine add-on, 7 patients (70.0%) experienced at least one adverse event (AE) (see Table 3). HAM-D mean scores decreased from 23.9 ± 3.9 (baseline) to 14.8 ± 5.3 (4 weeks) (Figure 1). 7/10 patients were responders at the end of period, of them 1 patient reached responder status by week 2. No patient met the criteria for remission. Dosage of cariprazine was increased to 3 mg/d in 4 patients. Table 3 summarizes dosage, timing of response and reported AEs in the sample of 10 patients.

**Table 1:** Demographic and clinical characteristics of the sample.

Parameters		*N* = 10
Age, years (mean ± SD)		52.3 ± 6.2
Sex, *n* (%)	Male Female	4 (40.0) 6 (60.0)
Marital status, *n* (%)	Single Married	1 (10.0) 9 (90.0)
Educational level, years (mean ± SD)		11.3 ± 3.4
Working for pay, *n* (%)	Yes No	5 (50.0) 5 (50.0)
Age at onset, years (mean ± SD)		25.4 ± 4.1
Number of episodes, (mean ± SD)		3.6 ± 1.3
Suicide attempts lifetime, *n* (%)	Yes No	4 (40.0) 6 (60.0)
Psychiatirc comorbidities, *n* (%)	Yes No	6 (60.0) 4 (40.0)
Type of psychiatric comorbities, *n* (%)	OCD Anxiety disorders SUD	3 (30.0) 3 (30.0) 2 (20.0)
Class of antidepressant, *n* (%)	SSRI SNRI TCA	5 (50.0) 2 (20.0) 3 (30.0)
Previous augmenting AA, *n* (%)	Aripiprazole Brexpiprazole Risperidone	4 (40.0) 3 (30.0) 3 (30.0)
HAM-D scores, (mean ± SD)		23.9 ± 3.9

AA: Atypical Antipsychotic; HAM-D: Hamilton Depression Rating Scale; OCD: Obsessive-Compulsive Disorder; SUD: Substance Use Disorder; SSRI: Selective Serotonin Reuptake Inhibitor; SNRI: Serotonin and Norepinephrine Reuptake Inhibitor; TCA: Tricyclic Antidepressant.

**Table 2:** Duration of of depressive episode, antidepressant treatment and dosage in the sample.

Patient	Duration of depressive episode (weeks)	AD combined with cariprazine	Daily dosage of AD during the add on
1	8	Fluvoxamine	300 mg
2	10	Sertraline	200 mg
3	11	Fluvoxamine	300 mg
4	12	Clomipramine	225 mg
5	12	Duloxetine	60 mg
6	14	Clomipramine	300 mg
7	24	Duloxetine	90 mg
8	15	Paroxetine	60 mg
9	22	Clomipramine	225 mg
10	28	Sertraline	200 mg

AD: antidepressant.

**Table 3:** Response, timing and adverse events in the sample.

Patient	Ham-D score			Responder	Final CPZ dose Mg/day	AEs
	Baseline	2 weeks	4 weeks			
1	16	17	18	No	3	Akathisia
2	20	20	21	No	3	–
3	24	14	12	Within week 4	1.5	–
4	29	18	14	Within week 4	1.5	Nausea
5	21	11	8	Within week 4	3	Tremor
6	24	12	9	Within week 2	1.5	Headache
7	26	23	23	No	3	Agitation
8	27	21	10	Within week 4	1.5	Xerostomia
9	27	18	13	Within week 4	1.5	–
10	25	23	20	No	3	Xerostomia

HAM-D: Hamilton Depression Rating Scale; CPZ: cariprazine; AEs: Adverse events.

## Discussion

To the best of our knowledge this is the first study focusing on the efficacy and tolerability of cariprazine as add-on agent in TRD real-world patients who failed a previous trial of AA augmentation of their AD therapy. Treating TRD is a clinical challenge due to its cost in terms of continuing disability, consequence for patients’ functioning and quality of life as well as resource utilization (1, 2, 28).

Although not licensed in all countries, cariprazine is one of the so called third generation antipsychotics that showed evidence in treatment of depression. In a phase 2 study flexible –dose cariprazine in adults with MDD and inadequate response to ongoing AD treatment, change from baseline to week 8 in Montgomery-Asberg Depression Rating Scale (MADRS) total score was significantly greater with cariprazine 2–4.5 mg/day compared with placebo (19). In a more recent phase 3 study adjunctive 1.5 mg/day of cariprazine demonstrated efficacy in reducing depressive symptoms in adults with MDD and inadequate response to AD alone (21). Although unipolar and bipolar depression are distinct illnesses, previously published bipolar studies showed positive results with cariprazine add-on (29–31) also when added to mood stabilizers and AD in patients with resistant bipolar depression (32). Collectively these studies support the efficacy of adjunctive cariprazine in reducing depressive symptoms. Our preliminary results show that cariprazine can reduce depressive symptoms in real-world TRD patients in the short-term period also in the sub-population of patients that already failed a first augmentation trial with another AA (in our sample risperidone, aripiprazole or brexpiprazole). At the end of the 4 weeks of observation seven out of ten patients met the criteria for a clinical response, one patient showed response already at week 2, However exiguity of the sample and descriptive nature of our study do not allow a comparison with literature about cariprazine add on. In Durgam et al. (19) rate of responders according to MADRS scores was 48% with cariprazine 1–2 mg/day and 50% with cariprazine 2–4,5 mg/day. In Sachs et al. (21) responders to cariprazine 1.5 mg/day added to ongoing AD therapy were 40.9 and 41% when dosage was 3 mg/day. In our sample most patients responded to a dosage of cariprazine of 1.5 mg/day. These data are congruent with previous observation that lower dose of this antipsychotic seem to be more effective in reducing depressive symptoms (21). In our sample there was no drop-out due to adverse events and there was no severe adverse event reported. In our samples cariprazine was associated with favorable tolerability profiles, low discontinuation rates as previously observed in other study (21). It should be noted that no patients of our study discontinued the previous AA added as augmenting agent, due to side effects but only to lack of efficacy.

In conclusion, our case series suggests that adding low dose cariprazine to AD therapy in TRD patients who failed a previous AA augmentation trial could be an efficacious strategy to ameliorate depressive symptoms and this seems to be true also in real-world patients with other psychiatric comorbidities. To the best of our knowledge this is the first observation in this direction. Our results suffer for several limitations, first the retrospective observational nature of the study and the exiguity of the sample. Further confirmation in larger population and in prospective studies is needed.

## Data availability statement

The raw data supporting the conclusions of this article will be made available by the authors, without undue reservation.

## Ethics statement

Ethical approval was not required for the study involving humans in accordance with the local legislation and institutional requirements. Written informed consent to participate in this study was not required from the participants or the participants’ legal guardians/next of kin in accordance with the national legislation and the institutional requirements. Written informed consent was obtained from the individual(s) for the publication of any potentially identifiable images or data included in this article.

## Author contributions

EP: Conceptualization, Investigation, Writing – original draft, Writing – review & editing. AM: Conceptualization, Investigation, Writing – original draft, Writing – review & editing. FR: Conceptualization, Investigation, Writing – original draft, Writing – review & editing. VM: Conceptualization, Investigation, Writing – original draft, Writing – review & editing.
